# Whole genome sequencing identified genomic diversity and candidated genes associated with economic traits in Northeasern Merino in China

**DOI:** 10.3389/fgene.2024.1302222

**Published:** 2024-01-25

**Authors:** Wenfeng Yi, Mingyue Hu, Lulu Shi, Ting Li, Chunyan Bai, Fuliang Sun, Huihai Ma, Zhongli Zhao, Shouqing Yan

**Affiliations:** ^1^ College of Animal Science, Jilin University, Changchun, China; ^2^ College of Agriculture, Yanbian University, Yanji, China; ^3^ Institute of Animal Husbandry and Veterinary, Jilin Academy of Agricultural Sciences, Gongzhuling, China

**Keywords:** Northeast Merino, whole-genome sequencing, genetic diversity, population structure, selection signatures

## Abstract

**Introduction:** Northeast Merino (NMS) is a breed developed in Northeast China during the 1960s for wool and meat production. It exhibits excellent traits such as high wool yield, superior meat quality, rapid growth rate, robust disease resistance, and adaptability to cold climates. However, no studies have used whole-genome sequencing data to investigate the superior traits of NMS.

**Methods:** In this study, we investigated the population structure, genetic diversity, and selection signals of NMS using whole-genome sequencing data from 20 individuals. Two methods (integrated haplotype score and composite likelihood ratio) were used for selection signal analysis, and the Fixation Index was used to explore the selection signals of NMS and the other two breeds, Mongolian sheep and South African meat Merino.

**Results:** The results showed that NMS had low inbreeding levels, high genomic diversity, and a pedigree of both Merino breeds and Chinese local breeds. A total length of 14.09 Mb genomic region containing 287 genes was detected using the two methods. Further exploration of the functions of these genes revealed that they are mainly concentrated in wool production performance (*IRF2BP2*, *MAP3K7*, and *WNT3*), meat production performance (*NDUFA9*, *SETBP1*, *ZBTB38,* and *FTO*), cold resistance (*DNAJC13*, *LPGAT1*, and *PRDM16*), and immune response (*PRDM2*, *GALNT8*, and *HCAR2*). The selection signals of NMS and the other two breeds annotated 87 and 23 genes, respectively. These genes were also mainly focused on wool and meat production performance.

**Conclusion:** These results provide a basis for further breeding improvement, comprehensive use of this breed, and a reference for research on other breeds.

## 1 Introduction

As one of the first domesticated livestock, sheep (*Ovis aries*) have contributed significantly to the development of human society by providing various products such as wool, milk, and meat. It is widely believed that domestic sheep originated from the Asiatic mouflon (*Ovis orientalis*) in Anatolia about 11,000 years ago, and have been dispersed to different parts of the world with human activities (Chen et al., 2021; Cheng et al., 2023). During the migration process, sheep around the globe faced diverse natural and artificial selection pressures, resulting in more than 1,400 breeds with significant differences (Diamond, 2002). There are 42 unique native breeds in China, which are often used to cross with exotic breeds to develop new breeds with high productivity (Wei et al., 2015). Northeast Merino (NMS), also known as Northeast Fine-wool, is the second wool breed successfully bred in China. It was developed in the 1960s in Northeast China from a cross between the Mongolian and Merino breeds as a dual-purpose breed for wool and meat (Yin et al., 1965). This breed has many advantages, such as high wool production, good meat quality, fast growth rate, strong disease resistance, and adaptation to cold environments (J, 2021). Consequently, NMS is popular and widely farmed.

Whole-genome sequencing (WGS) technology can discover a large number of variants that can be used as molecular genetic markers. This is an important method for studying the origin and domestication of species, animal breeding, candidate genes for economically important traits, and so on. This method is widely used to explore the genomic characteristics of various species and has obtained many important results in the field of animal husbandry (Weigend and Romanov, 2002; Zhang et al., 2022). Studies based on WGS have identified some genes related to important economic traits in sheep. Shi et al. identified several genes involved in growth, development, and high-altitude adaptation by studying the selection signal of Panou Tibetan sheep (Shi et al., 2023). Cheng et al. studied gene flow from wild to domesticated sheep and found candidate genes related to morphology and adaptation (Cheng et al., 2023).

However, few studies have been conducted on NMS and they mainly focus on production performance and breeding improvement, with no reports on the genome-wide genetic characteristics of NMS (Huo et al., 2022). To increase understanding of NMS genomic variation and discover candidate regions associated with its superior characteristics, WGS was performed on 20 NMS for the first time in this study. By combining sequencing data for 177 published individuals from 11 other breeds, the population structure, genetic diversity, and selection signals of this breed were explored. Our results will lay the foundation for further research on the economically important traits of NMS, offer guidance for future breeding and utilisation, and provide a reference for research on other improved breeds.

## 2 Materials and methods

### 2.1 Sample collection and sequencing

Genomic DNA was extracted using the EasyPure Blood Genomic DNA Kit (TransGen Biotech) from blood samples collected of Northeast Merino rams (NMS, n = 20) from Jilin Qianyang Agriculture and Animal Husbandry Co., Ltd.(Songyuan City, Jilin Province, China). For each individual, 2 × 150 bp paired-end read data were sequenced using DNBSEQ-T7 at Novogene Bioinformatics Institute company (Beijing, China) (Supplementary Table S1). In addition, to better study the population structure and selection signals of NMS, WGS data for 177 published sheep of 11 breeds were obtained from the Sequence Read Archive (https://www.ncbi.nlm.nih.gov/sra/). Including South African Meat Merino (SAM, n = 10), Australian Merino (AMS, n = 25), Rambouillet (RAM, n = 10), Chinese Merino (CMS, n = 20), Dorset (DOR, n = 24), Suffolk (SUF, n = 12), and several Chinese local breeds: Hu (HUS, n = 10), Small-tailed Han (STH, n = 21), Altay (ALT, n = 10), Tibetan (TIB, n = 12), and Mongolian (MON, n = 23) (Supplementary Table S2).

### 2.2 Reads mapping and variant identification

Burrows-Wheeler Aligner (BWA) software (v0.7.13) was used for mapping clean reads from all 197 sheep to the *Ovis aries* reference genome Oar_rambouillet_v1.0 (https://www.ncbi.nlm.nih.gov/datasets/genome/GCF_002742125.1/) using ‘bwa mem’ parameters (Li and Durbin, 2009). And Picard MarkDuplicates tool (v1.115) (https://github.com/broadinstitute/picard) was used to remove duplicate reads from each alignment. GATK (v4.1.4) was used for SNP calling and then the results were filtered with GATK’s “VariantFiltration” module (McKenna et al., 2010). The filtering parameters were ‘QD < 2.0 ||FS > 60.0 || MQ < 40.0 || SOR > 3.0 || MQRankSum < −12.5 ||ReadPosRankSum < −8.0'. In addition, bcftools (v1.8) was used to extract the biallelic loci located on the autosomes from the hard filtered results, and PLINK software (v1.9) for quality control with parameters’--geno 0.05 --mind 0.1 --maf 0.03’ (Purcell et al., 2007; Danecek et al., 2021).

Based on the annotation file of the Oar_rambouillet_v1.0 reference genome, the types of each SNP were annotated by SnpEff software (v5.1d) (Cingolani et al., 2012). Using previously reported methods, the genes with more than five NMS-specific non-synonymous variations were further extracted for analysis (Kawahara-Miki et al., 2011). To better understand the function of these genes, DAVID was used for online Gene Ontology (GO) and Kyoto Encyclopedia of Genes and Genomes (KEGG) enrichment analysis (Huang da et al., 2009; Sherman et al., 2022). Pathways with a *p*-value less than 0.05 were considered significantly enriched.

### 2.3 Population genetic analysis

Nucleotide diversity (*pi*), expected heterozygosity (*H*_E_), observed heterozygosity (*H*_O_), linkage disequilibrium (LD) decay, and the runs of homozygosity (ROH) for all breeds were calculated and analysed to explore the genomic genetic diversity of NMS. The values of *pi* were calculated by VCFtools software (v0.1.16) with the parameters ’--window-pi 10,000 --window-pi-step 5,000' (Danecek et al., 2011). PLINK software was used to calculate *H*_O_ and *H*_E_ with the ‘--hardy’ parameter. The number and length of ROH for each individual were calculated with the ‘--homozyg-density 10 --homozyg-gap 100 --homozyg-kb 100 --homozyg-snp 10 --homozyg-window-het 1 --homozyg-window-missing 5 --homozyg-window-snp 50 --homozyg-window-threshold 0.05’ parameter of PLINK software (v1.9). Based on the analysis of ROH, the genomic inbreeding coefficient was calculated using the following formula: *F*_ROH_ = ∑*L*_ROH_/*L*_AUTO_, where *L*_ROH_ is the total length of ROH fragments per individual and *L*_AUTO_ is the total length of autosomes covered by SNPs sequenced across the genome. LD decay was calculated using the PopLDdecay software (v3.42) with the ‘-MaxDist 1,000’ parameter (Zhang et al., 2019).

### 2.4 Phylogenetic and population structure analysis

After quality control, PLINK software with the ‘--indep-pairwise 50 5 0.2’ parameter was used to remove high LD sites in the dataset. The obtained sites were used for population structure analysis. GCTA software (v1.92.3) was used to perform principal component analysis (PCA) with the parameter ‘grm’ (Yang et al., 2013). Using the pairwise genetic distances matrix calculated by PLINK, a phylogenetic tree was constructed based on the neighbor-joining (NJ) model by MEGAX and visualised with iTOL (https://itol.embl.de/, accessed on 21 June 2023) (Kumar et al., 2018; Letunic and Bork, 2021). ADMIXTURE software (v1.3.0) was used to infer ancestral populations with K = 2–6. For each K, the software was run 10 times with random number seeds and chose the result with the lowest average cross-validation (CV) error (Alexander et al., 2009).

### 2.5 Identification of selection signature

Two methods, integrated haplotype score (iHS) and composite likelihood ratio (CLR) were used to detect regions of the genome subject to selection in the NMS population. For SNPs detected in the NMS population, BEAGLE (v5.4) was used for imputing and phasing genotypes (Browning and Browning, 2009), and selscan software (v1.2) was used to calculate iHS (Szpiech and Hernandez, 2014). The results were normalised by the norm module of selscan with a window size of 50 kb, the final score for each window is calculated based on the number of SNPs in the window with an iHS score greater than 2 in absolute values. In conclusion, the top 5% of the windows with the highest final scores were retained as the candidate area subject to selection. CLR was calculated by SweeD software (v4.0.0) within a non-overlapping 50 kb window, the top 5% of windows with the highest CLR values are regarded as candidate selected areas (Pavlidis et al., 2013). Only candidate regions that were determined by both methods were considered to be under positive selection. To study the unique selection signals of NMS in recent years and their genetic divergence from other breeds, VCFtools software (v0.1.16) was used to calculate the Fixation Index (*F*_ST_) between NMS and two other breeds with a non-overlapping 50 kb window. The MON is an established parental breed of NMS, while the SAM was introduced to improve the meat performance of NMS (Yang et al., 2017). The 5% of the window with the highest value in the *F*_ST_ calculation was considered candidate areas, where regions detected by all three methods were considered to be regions with selection differences between breeds. The results of the selected signals are analyzed using SnpEff software (v5.1d) and DAVID in the same way as above.

### 2.6 Functional annotation based on the QTL database

The sheep quantitative trait locus database (Sheep QTLdb) contains previously reported regions of QTLs and association data associated with important production traits in sheep (Hu et al., 2022). To calculate the main function of the selected area, the results of the selected signals were compared with the sheep QTLdb (published 25 April 2023).

## 3 Results

### 3.1 Whole-genome sequencing and SNP detection

Using the DNBSEQ-T7 platform, 682.4 Gb of raw data was obtained from 20 NMS individuals and the details of the sequencing data are given in Supplementary Table S1. After filtering, 673.01 Gb clean data were retained, and individual genomes of NMS were generated with an average depth of ∼11.90×. After quality control, 27,770,572 high-quality autosomal biallelic SNPs were obtained. In brief, there were 46,860,074 SNPs before quality control, of which a total of 2,423,764 SNPs were removed using the ‘geno 0.05’ parameter, no individuals were removed due to ‘mind 0.1’ and 16,665,738 SNPs removed by ‘maf 0.03’. Among the remaining SNPs, there are a total of 19,833,469 transitions (Ts) and 7,937,103 transversions (Tv) in all SNPs, with a Ts/Tv ratio of 2.50.

In addition, a total of 23,975,257 high-quality SNPs in 20 NMS were detected. Most of the variants were located in intergenic (54.33%) and intronic regions (35.61%), and only 0.62% (including 47,910 non-synonymous variants and 99,926 synonymous variants) were located in exons (Supplementary Table S3). Of the NMS-specific 1,975,256 SNPs, 53.29% and 34.82% of the variants were located in intergenic and intron regions, respectively. The numbers of non-synonymous and synonymous variants were 8,673 and 11,383, respectively, accounting for 0.44% and 0.58% of the total (Supplementary Table S4).

### 3.2 Functional enrichment analysis of the specific SNPs in NMS

Non-synonymous SNPs specific to NMS were annotated using SnpEff software, resulting in 2,864 genes. Of these, 350 genes containing more than five non-synonymous variants were selected for enrichment analysis. A total of 11 GO terms were significantly enriched (*p* < 0.05), of which the most significant (*p* = 0.002653) GO term was “calcium ion binding, GO:0005509”, containing 13 genes. Several biological process terms were related to immunity, such as “antigen processing and presentation of peptide or polysaccharide antigens via MHC class II, GO:0002504”, “antigen processing and presentation, GO:0019882” (Supplementary Table S5). In addition, “heat shock protein binding, GO:0031072”, a molecular function term related to heat shock protein binding activity was enriched (Yue et al., 2020). For KEGG, 13 pathways were significantly enriched (*p* < 0.05), of which the most significant (*p* = 2.80E-09) was “Graft-versus-host disease, oas05332”, associated with immune response. Moreover, several pathways related to immunity and disease, such as “Antigen processing and presentation, oas04612”, and “Phagosome, oas04145” were also enriched. Notably, the pathway “Hippo signalling pathway - multiple species, oas04392”, which is associated with a wide range of important production traits was enriched (Supplementary Table S5) (Deng et al., 2016; Deng et al., 2019; Yatsenko et al., 2020; Dos Santos et al., 2022).

### 3.3 Population structure and relationships

To investigate the population relationship between NMS with other breeds, the 2,330,031 SNPs after Linkage pruning, was used for admixture analysis, phylogenetic analysis and PCA.

**FIGURE 1:**
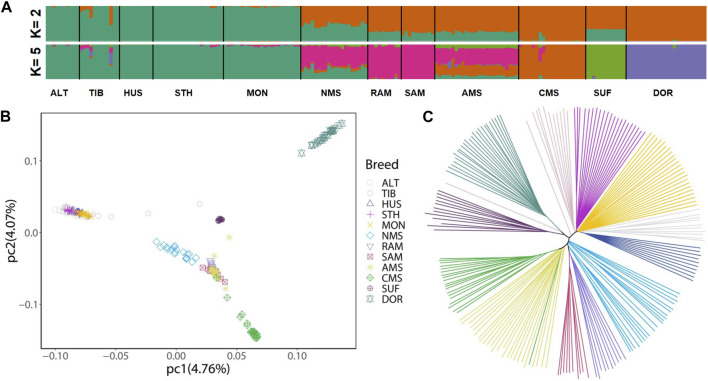
Population structure and relationships of Northeast Merino compared with other breeds. **(A)** Admixture plot of 12 sheep breeds using ADMIXTURE with K = 2 and K = 5. **(B)** Principal component analysis of 12 sheep breeds. **(C)** A Neighbor-joining phylogenetic tree of the 12 sheep breeds (197 animals). Abbreviations: ALT, Altay; TIB, Tibetan; HUS, Hu; STH, Small-tailed Han; MON, Mongolian; NMS, Northeast Merino; RAM, Rambouillet; SAM, South African Meat Merino; AMS, Australian Merino; CMS, Chinese Merino; SUF, Suffolk; DOR, Dorset.

The results of ADMIXTURE showed that when K = 2, the ancestors of the China local breeds had a single genomic composition, while Merino breeds showed a mixed ancestral component except for CMS. And when K = 5, NMS displayed clear evidence of shared genome ancestry with the Merino breeds (average 55.00%) and China local breeds (average 30.11%) (Figure 1A). For PCA, the genetic data variation was explained by 4.76% and 4.07% of the first and second principal components, respectively. The results showed that Chinese local breeds and the three Merino breeds (RAM, SAM, and AMS) were clustered separately, with CMS forming a distinct cluster. And NMS was located between Chinese local breeds and the three Merino breeds (Figure 1B). The NJ tree also revealed the same pattern of NMS located between Chinese local breeds and Merino breeds (Figure 1C).

### 3.4 Genetic diversity analysis

To compare the distribution of ROH fragments in different breeds, ROH fragments were classified into five categories according to their length (0–0.5 Mb, 0.5–1 Mb, 1 Mb–2 Mb, 2 Mb–3 Mb, >3 Mb). Most ROH lengths were in the range of 0–0.5 Mb in all breeds, with only TIB, SUF, RAM, and SMA detecting ROH fragments greater than 3 Mb in length (Supplementary Table S6).

**FIGURE 2:**
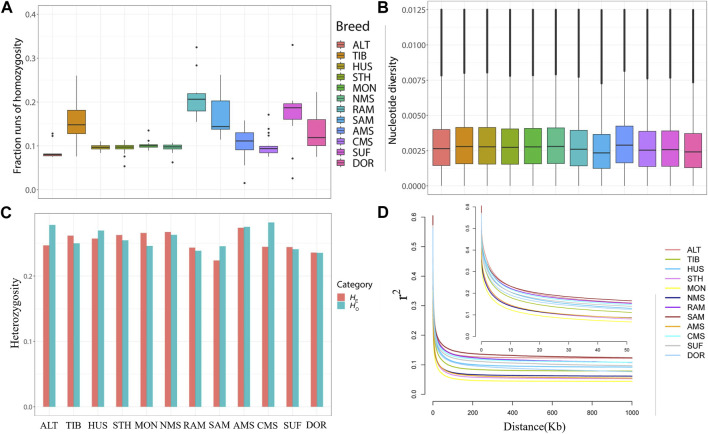
Summary statistics for genetic diversity. **(A)** Box plots of genomic inbreeding coefficient for each breed. **(B)** Nucleotide diversity of each breed across the genome in windows of 50 kb with steps of 50 kb. **(C)** The distribution of expected heterozygosity (*H*_E_) and observed heterozygosity (*H*_O_) in each breed. **(D)** Linkage disequilibrium (LD) decay in the 12 sheep breeds of China, with a line for each breed.

The total ROH length of NMS is medium, lower than the four Merino breeds and the two commercial breeds which have been subjected to stronger selection pressure. *F*_ROH_ results showed that NMS had a low inbreeding coefficient (0.095732) and ranked 10th in 12 breeds, higher than ALT (0.088212) and STH (0.094908) only, while RAM had the highest inbreeding coefficient (0.213313). (Figure 2A, Supplementary Table S7). In terms of nucleotide diversity, NMS was ranked 4th (0.002971) behind HUS (0.002979), TIB (0.002988), and AMS (0.003066), while SAM had the lowest *pi* value (0.002590) (Figure 2B and Supplementary Table S7). Similarly, NMS also exhibited a high level of heterozygosity with the *H*_O_ (0.262932) and *H*_E_ (0.267285) values, ranking fifth and second, respectively (Supplementary Table S7, Figure 2C). Regarding LD decay, the results were similar to those of *F*_ROH_. The r^2^ values of all breeds decreased rapidly with increasing genomic distance, with the fastest decrease in the first 50 kb. For the distance between markers that was greater than 50 kb, the results showed that NMS had a low genome-wide LD, ranking ninth out of 12 breeds, with MON showing the lowest LD and SAM showing the highest (Figure 2D).

### 3.5 Genomic selection signatures analysis

**FIGURE 3:**
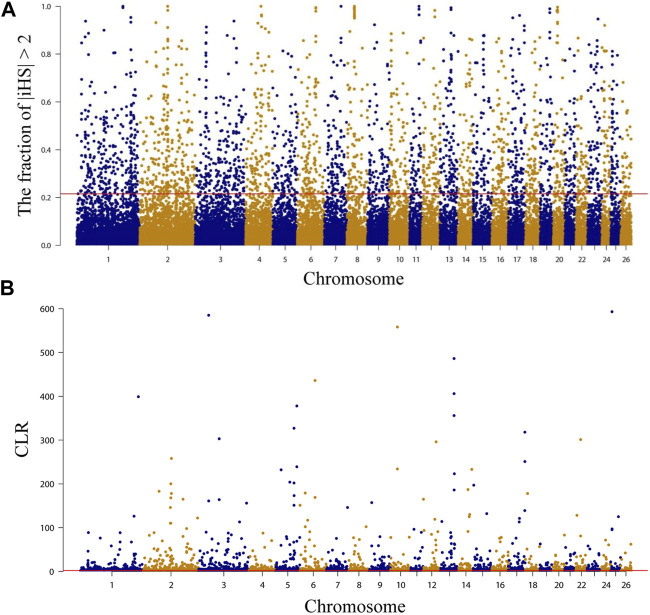
Characterization of positive selection in the genome of Northeast Merino. The red line is the 5 percent threshold. **(A)** Manhattan plots of selection sweep results for iHS in Northeast Merino. **(B)** Manhattan plots of selection sweep results for CLR in Northeast Merino.

Selected regions on the NMS genome were examined using both integrated haplotype score (iHS) and composite likelihood ratio (CLR) methods, and the top 5% of each method was extracted for annotation. A total of 2,039 (Figure 3A, Supplementary Table S8) and 2,221 (Figure 3B, Supplementary Table S9) genes were annotated by iHS and CLR, respectively, and 14.09 Mb of chromosomal regions containing 287 genes were detected by both methods (Supplementary Table S10). Overall, a range of candidate genes associated with economical traits was subject to positive selection, such as wool growth and type regulation (*IRF2BP2*, *GLI2*, and *PKIG*) (Harmon and Nevins, 1997; Mill et al., 2003; Demars et al., 2017; Zhang et al., 2020; Lv et al., 2022), hair follicles development (*RBM28*, *MAP3K7*, and *WNT3*) (Millar et al., 1999; Sayama et al., 2006; Sayama et al., 2010; Warshauer et al., 2015), growth and development-related (*NDUFA9*, *SETBP1*, and *ZBTB38*) (Liu et al., 2010; Khansefid et al., 2018; Zlobin et al., 2021), meat quality and fat deposition (*CTCF*, *PARP4*, and *USP25*) (Rouleau et al., 2010; Hamill et al., 2012; Yuan et al., 2022), cold resistance (*DNAJC13*, *LPGAT1*, and *PRDM16*) (Shi and Manley, 2007; Seale et al., 2008; Lynes et al., 2018; Liu et al., 2022a), and immune-related (*PRDM2*, *GALNT8*, and *HCAR2*) (Al Kalaldeh et al., 2019; Wang et al., 2019; Ghoreishifar et al., 2020), etc. Subsequently, GO and KEGG enrichment analyses were performed on the 287 genes using DAVID. The results showed that KEGG was enriched to only two pathways, but neither was significantly enriched. One of them was “NOD-like receptor signaling pathway, oas04621” (*p* = 0.052668), containing 6 genes (*NLRP12*, *DNM1L*, *MAP3K7*, *LOC101103623*, *LOC101103376*, and *LOC101105481*) and is associated with immunity (Van Gorp et al., 2014). The other was “*Yersinia* infection, oas05135”(*p* = 0.078788), containing 5 genes (*MAP3K7, PXN*, *DOCK1*, *LOC101103376*, and *LOC101103623*) and is also associated with immunity (Supplementary Table S10). Regarding GO enrichment analysis results, the most significant term (*p* = 0.005476) was “acute-phase response, GO:0006953” (*LOC101120204, LOC101120613*, and *LOC105601867*), which is related to immunity (Pannen and Robotham, 1995). In addition, another two highly significant enrichment (*p* < 0.01) terms may be related to meat quality. “actomyosin structure organization, GO:0031032” contains 3 genes (*EPB41L4B*, *CDC42BPB*, and *CDC42BPA*), which is related to composition and disassembly of structures made up of actin and myosin or paracrine. Among them, *CDC42BPB* may affect the synthesis of 3-hydroxybutyric acid and thus the quality of meat (Li et al., 2022). And “high-density lipoprotein particle, GO:0034364” (*LOC101120204*, *LOC101120613*, and *LOC105601867*) (Supplementary Table S11), which is a cellular component term involved in the transport of lipids (Zhao et al., 2022b).

Annotation of candidate regions detected by both methods using the Sheep QTLdb to determine the function of selected genomic regions in the NMS population. The results showed that 361 QTLs were detected in 420 non-overlapping candidate regions (Supplementary Table S12). The largest proportion of these QTLs was related to meat and carcass, with 113 QTLs (31.30%) distributed across 290 candidate regions. Health-related QTLs followed, with a total of 73 QTLs (20.22%) detected in 217 regions. In addition, 19 wool-related QTLs (5.26%) were also detected, distributed across 135 regions. This suggested that NMS was strongly selected for meat and wool production traits during the breeding process.

Based on *F*_ST_, the selection signals between NMS and other breeds were explored. For MON, 2,389 genes (Supplementary Table S13) were annotated and 3.75 Mb of chromosomal regions contained 87 genes (Supplementary Table S14) that were also detected by iHS and CLR. In contrast to within-population selection signals, the genes detected were mainly related to hair production traits and meat-production traits, such as *IRF2BP2* (Lv et al., 2022), *SETBP1* (Khansefid et al., 2018), and *LNX2* (Santana et al., 2015). In addition, *PRDM16* (Liu et al., 2022a), which is related to cold resistance, was also detected. Enrichment analyses were performed of these 87 genes using DAVID and 14 terms and 12 pathways were significantly enriched (Supplementary Table S15). The two most significant terms were “D-threo-aldose 1-dehydrogenase activity, GO:0047834” (*p* = 0.000007) and “synaptic transmission, glutamatergic, GO:0035249” (*p* = 0.001591), related to lipid accumulation (Poorinmohammad et al., 2022) and wool colour, respectively (Zhang et al., 2023). The most significant pathway was the “Citrate cycle (TCA cycle), oas00020” (*p* = 0.002265), which contains five genes related to metabolism. In the *F*_ST_ results between NMS and SAM, 2,103 genes (Supplementary Table S16) were annotated in the candidate regions. Among them, 1.58 Mb of chromosomal regions contained 23 genes (Supplementary Table S17) that were detected by all three methods. The annotated genes were mainly divided into two categories: immunity and disease resistance, such as *GALNT8*(Al Kalaldeh et al., 2019), and *RASAL2* (Mesure et al., 2010); and body size, such as *NDUFA9* (Khansefid et al., 2018), *ADGRD1* (Fischer et al., 2016). Enrichment analysis was performed on these 23 genes and one term and three pathways that were significantly enriched (Supplementary Table S18). The most significant pathway was “Chemical carcinogenesis - reactive oxygen species, oas05208” (*p* = 0.020840), which might be related to environmental adaptation (Li et al., 2017). The only term was “D-threo-aldose 1-dehydrogenase activity, GO:0047834” (*p* = 0.024574), which was also significantly enriched in the selection signal results with MON.

## 4 Discussion

Understanding the genetic diversity of breeds allows a sound assessment of their status and is important for using and conserving genetic resources. Generally, the higher the intensity of selection on a breed, the lower its genetic diversity and the greater the coverage of runs of homozygosity (Lv et al., 2022). Among these 12 breeds, NMS had a relatively high genetic diversity, as indicated by its high values of *pi*, *H*_O_, and *H*_E_. Similarly, *F*_ROH_ values and LD decay of the NMS also support this view, both of which are at low levels. Regarding ROH, long ROH arises from inbreeding, whilst shorter ROH reflects the effect of remote ancestors (Purfield et al., 2012). The majority of fragments detected in NMS were between 0 and 0.5 MB in length, with no fragments over 3 MB in length detected, the distribution pattern of ROH fragments was generally consistent with previous reports (Supplementary Table S6) (Cheng et al., 2020). These findings indicate that NMS had high genetic diversity and a low degree of inbreeding, which may also be related to the fact that it was recently bred in the 1960s and had not been subjected to strong long-term selection (Yin et al., 1965). In addition, the higher genetic diversity also means that NMS has great breeding potential and is an excellent breed for further selection.

The results of admixture analysis, NJ tree, and principal component analysis (PCA) all confirmed that the NMS was bred by crossbreeding Chinese local breeds and Merino breeds. According to the admixture analysis results (Figure 1A), when K = 5, the main sources of ancestral components of NMS were Merino breeds (average 55.00%) and Chinese local breeds (average 30.11%), indicating that Merino breeds more influenced NMS during the breeding process. Regarding the PCA results, it is worth noting that the NMS population was more dispersed in the cluster., which reflects the possibility of greater genetic variation among NMS individuals.

To explore NMS-specific superior traits, genes containing more than five NMS-specific non-synonymous SNPs were selected for enrichment analysis. The results showed highly significant enrichment (*p* < 0.01) for multiple GO terms and KEGG pathways associated with immunity and disease (Supplementary Table S5). Additionally, GO enriched to “heat shock protein binding, GO:0031072”, which is associated with heat shock protein binding activity (Yue et al., 2020). It has been reported that the expression of heat shock proteins is increased in mice exposed to cold stimuli (Liu et al., 2022b). This phenomenon has also been observed in goats and the expression of the heat shock protein 70 gene is breeds specific (Banerjee et al., 2014). Therefore, “heat shock protein binding” may be related to the adaptation of NMS to the environment of northeastern China, which is known for its long and cold winters. In terms of production performance, the pathway “Hippo signaling pathway - multiple species, oas04392” was enriched. Hippo signalling has very important biological functions, such as cell proliferation (Deng et al., 2016), muscle development (Yatsenko et al., 2020), follicular growth and development (Dos Santos et al., 2022), adipogenesis (Deng et al., 2019) and hair follicle development (He et al., 2022). These genes may be related to the germplasm characteristics of NMS and their influence on the NMS phenotype still needs to be further explored.

Selection scanning was also performed in NMS and the candidate regions detected by both methods contained a total of 287 genes. As an excellent breed for both meat and wool, the NMS has been extensively bred and farmed in northeast China for the past 50 years (Long, 2019), leading to further improvements in NMS production performance. Therefore, the functions of these genes were explored to understand selection pressure better.

For breeders, the productive performance of the livestock is the primary concern. In the selected region of NMS, several genes have been reported to be associated with wool production performance, such as *GLI2*, which is a key mediator of Sonic hedgehog (Shh) signalling, that mediates the mitogenic action of Shh to regulate the density of wool and hair follicles (Mill et al., 2003; Zhang et al., 2020). *IRF2BP2*, which differs significantly between coarse and fine-wool sheep, is thought to regulate coarse and fine wool by affecting the expression of *VEGFA* (Demars et al., 2017; Lv et al., 2022). *WNT3*, which plays an important role in hair follicle development (Millar et al., 1999). With regard to meat production performance, several previously reported genes were also detected. *FTO*, which has been reported to be associated with a variety of fat-related traits in animals (Chang et al., 2018), especially, is linked with tail fat deposition in Hu sheep (Zhao et al., 2022a). *CTNNBL1* (Yin et al., 2012), and *SLIT2* (Mastrangelo et al., 2019; Ceccobelli et al., 2023) are also involved in fat deposition. *YARS2* was related to mitochondrial protein synthesis and mitochondrial respiration, the results of a genome-wide association analysis of Yorkshire pigs suggest that it might be associated with the feed conversion rate (Miao et al., 2021). In addition, two related terms were detected in the enrichment analysis, “actomyosin structural organization, GO:0031032” (*EPB41L4B*, *CDC42BPB*, and *CDC42BPA*) and “high-density lipoprotein particle, GO:0034364” (*LOC101120204*, *LOC101120613*, and *LOC105601867*), these genes may have a significant effect on the flesh quality of NMS.

Northeast China is renowned for its severe cold and NMS which is widely farmed in this region, possibly under positive selection for cold tolerance. Genome-wide selective scanning supports this hypothesis, as *DNAJC13* has been reported to be a key gene for cold resistance in Chinese white wax scale insects (Zhang et al., 2021). Moreover, several genes associated with brown fat, an important thermogenic tissue, were also identified (Cannon and Nedergaard, 2004). *PRDM16* can promote the formation of brown fat cells and the production of brown fat (Seale et al., 2008). *LPGAT1*, which is involved in the synthesis of cardiolipin and thus involved in the thermogenesis of brown fat (Lynes et al., 2018; Liu et al., 2022a). Immunity is also an important component and aspect of environmental adaptability and several candidate genes related to those were detected. The membrane-associated protein encoded by the *ABCB9* gene is associated with antigen processing (Fujimoto et al., 2011). *GALNT8* is related to innate and acquired immune responses and cytokine signalling, which are important for protecting sheep from parasitic invasion (Al Kalaldeh et al., 2019). These environmental adaptation-related genes may be important in enhancing the survival of NMS. Similarly, the QTL database test results also detected a high number of meat production-related, wool production-related and health-related QTLs.

It is worth noting that several genes with more than five breed-specific non-synonymous SNPs of the above among the selected candidates were identified. For example, *NLRP12*, which can suppress inflammation by negatively regulating NF-κB signalling, might be associated with the unique local environment (Wang et al., 2019). *PARP4* may be related to the unique fleshy traits of NMS, as it has been reported to be a very important role in the regulation of adipogenesis (Rouleau et al., 2010). The exploration of such genes will enhance the understanding and improvement of NMS characteristics and facilitate breeding other breeds in this region.

In addition, by comparing the selection signals between populations, the breed-specific selected regions and genes can be identified which reflects the evolutionary history and direction of the population. Both MON and SAM have been used to breed NMS, and their genomic differences can reveal the breeding objectives of NMS. In general, the breeds derived from local and commercial varieties are characterized by high adaptability and high production performance, as demonstrated by our experimental results. Compared to MON, the annotated genes were mainly focused on wool and meat production performance, such as *IRF2BP2* (Lv et al., 2022), and *SETBP1* (Khansefid et al., 2018). The two most significant terms in the enrichment analysis results, “D-threo-aldose 1-dehydrogenase activity, GO:0047834” (*p* = 0.000007) and “synaptic transmission, glutamatergic, GO:0035249” (*p* = 0.001591), are related to lipid accumulation and wool colour, respectively. On the other hand, compared to SAM, the annotated genes were mainly involved in immunity and somatic phenotypes, such as *GALNT8* (Al Kalaldeh et al., 2019), and *RASAL2* (Mesure et al., 2010). The most significant term in the enrichment results, “Chemical carcinogenesis - reactive oxygen species, oas05208” (*p* = 0.020840) was also associated with environmental adaptation and immunity. In addition, it is noteworthy that the selection signalling results with both MON and SAM were enriched to “D-threo-aldose 1-dehydrogenase activity, GO:0047834” and “Folate biosynthesis, oas00790”. The former is associated with fat accumulation, while the latter has no direct evidence of function in the literature, but folate is a vital vitamin that participates in various biological activities and has a crucial role in the immunity of living organisms (Lucock, 2000). This may mean that NMS has been subject to selection and breeding in recent years for meat production and immunity.

Generally, the genes that were subject to selection fall into four categories: wool-producing traits, meat-producing traits, immunity, and environmental adaptation. The specific molecular mechanisms and functions of these SNPs and genes may require subsequent experimental verification.

## 5 Conclusion

This study explored genomic diversity and selection models in Northeast Merino based on whole-genome sequencing data. The genomic diversity and population structure results reveal that NMS has high genomic diversity and shares genetic relationships with both Merino breeds and local Chinese breeds. Moreover, a range of candidate genes has been identified that may be important in the productive performance and environmental adaptation of this breed. These results lay a solid foundation for future breeding and also serve as a reference for other breeds.

## Data Availability

The datasets presented in this study can be found in online repositories. Sequencing reads of Northeast Merino have been submitted to NCBI with accession number PRJNA1002413.
